# Rational and practical use of imaging in COVID-19 pneumonia

**DOI:** 10.12669/pjms.36.COVID19-S4.2760

**Published:** 2020-05

**Authors:** Saba Sohail

**Affiliations:** Prof. Saba Sohail, MBBS, MCPS, FCPS, PhD, Professor of Radiology, Dow Medical College and DRKMP Civil Hospital, Chairperson, Department of Radiology, Dow University of Health Sciences Karachi, Pakistan

**Keywords:** Basildon protocol, COVID-19 Pneumonia, Chest X-ray, Contamination, CT scan, Disinfection, Fleischner Society statement, Portable Digital Radiography, Pakistan, proposed guidelines, Radiological Society of Pakistan, Ultrasound

## Abstract

The severe form of the COVID-19 pandemic caused by the *SARS-CoV-2* virus, has largely manifested as a predominant respiratory illness causing severe pneumonia characterized by bilateral, subpleural ground glass haze, progressing to consolidation, and fibrosis on imaging. There is some discrepancy between the governmental guidelines, professional Societies and Radiology and Respiratory Medicine specialists with divided opinions between the use of the chest X-rays and CT scan, and whether the use be screening or diagnostic. So far, the most balanced recommendations have been proposed by the Fleischner Society, which are endorsed by the Radiological Society of Pakistan as well. This writeup describes the approach for a rational use of imaging to the best advantage in the current situation according to local resources, and restricting the spread of infection. The most practical compromise for Pakistan appears to be the use of portable digital radiography equipment, and point-of- care ultrasound; with CT scan reserved for clinical situations not explained by the above two modalities, or demanding disease stratification.

## INTRODUCTION

**Fig.1:**
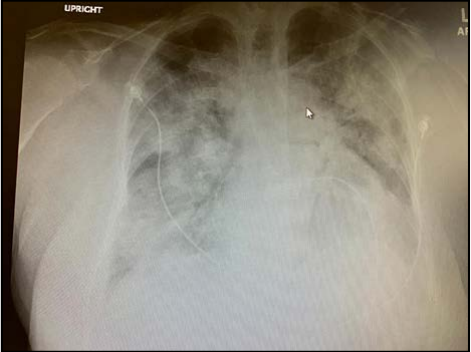
Chest x-ray of a PCR-positive adult with severe COVID-19 Pneumonia, showing extensive bilateral peripheral ground glass haze- (arrow), and consolidation (Image courtesy: Dr Imran Sharief, MD, FCCP)

**Fig.2:**
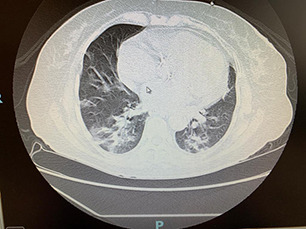
Non- contrast thin-section CT Chest in axial plane, taken at the level of middle and lower lobes, showing the combination of bilateral, asymmetrical, subpleural ground glass pattern, interstitial thickening and consolidation. The involvement is bilateral, and subpleural, with most lesions located in anatomical regions which would be hidden by the mediastinal structures and cardiac silhouette in a frontally projected chest radiograph. (Image courtesy: Dr Imran Sharief, MD, FCCP)

The COVID -19 disease is caused by the *SARS-CoV-2* virus. In most cases, this causes mild upper respiratory symptoms but the current pandemic has been made fearful by the unexpected virulence of the novel Corona virus causing a severe interstitial pneumonia in some cases, that rapidly transforms into an organizing pneumonia and causes an acute serious-to-fatal respiratory failure. This end of the disease spectrum has been shown to have specific radiological findings of bilateral, multifocal randomly scattered ground glass haze, in subpleural, mainly peripheral distribution with thickened pulmonary interstitium giving a reticular pattern, broncho-vascular prominence, and consolidation with increasing severity in the more seriously ill patients (figures 1 and 2).^1^,^2^ Enlarged lymph nodes are typically absent and pleural effusion is extremely rare. These findings are pretty much the same on CT scan and X-ray, the only difference being that CT picks the more subtle signs of disease because of its greater inherent spatial and contrast resolution detection range. Ultrasound basically detects the subpleural consolidation and rules out effusion and pneumothorax in emergency.^3^

The diagnosis of the disease is based on clinical symptoms and signs in an appropriate exposure setting, with positive PCR test for viral nucleic acid as the gold standard. However, the availability and the time required by the results are a major restriction in most parts of the world, including Pakistan. According to unpublished reports, Pakistan is testing about 5000 suspected cases daily, with 9% turning positive and requiring retesting in the next 14 days to be let out of quarantine and ensure non-infectivity. The number of kits is not sufficient to test the extrapolated high number of positive cases. On the other hand, diagnosed cases with moderate to severe clinical disease require monitoring and follow up for resolution or complications for day- to day care. This is where radiologic imaging steps in and its rationality and best utility needs to be assessed.

### Role of imaging in COVID-19 pneumonia

Radiology can play four basic roles- screening, diagnosis, severity assessment and monitoring the treatment response. The choice of radiologic modalities to fit in these roles is to be made from chest x-ray, chest CT scan (selecting from the range of non-contrast routine thin-section CT, dedicated HRCT, contrast enhanced CT scan, and dedicated CT pulmonary angiography or CTPA), and ultrasound (again to choose between regular portable or point-of care equipment). Each of these roles needs to be considered for the best-practice option.

***a)Screening:***

According to WHO, screening is done to detect potential health disorders or diseases in people who do not have any symptoms of disease. From this point of view, none of the above modalities fulfill the role despite many and contrary claims in favor and against, particularly for CT sanning.^4^ The reason is simple and logical. By the time abnormal findings appear on any of these modalities,^5^ the affected person is symptomatic no longer fitting in the WHO definition of detecting the asymptomatic.

***b)Diagnosis:***

This role is definite by virtue of the well described radiological signs of COVID -19 pneumonia and all the different modalities can be used for this purpose in a patient with suspected moderate to severe disease who has had symptoms for some time. As stated above, the abnormal findings are seen earlier on the CT scan than on the chest x-ray with greater accuracy.^5^ So, if a patient is having symptoms for a longer duration of time before coming to a health care facility, even the chest x-ray is good enough for diagnosing COVID pneumonia (Fig.1) and differentiating it from other cases of fever with dyspnea such as abscess, community acquired pneumonia or fungal or tuberculous disease.

Likewise, in emergency settings, even the non-radiologist emergency physician can perform a bed-side portable ultrasound only to see if there is a peripheral consolidation and whether there is an associated pleural effusion or not. Both these signs are diagnostic points of importance and affect the course of management in the present scenario. Needless to say, advanced technology with digital and portable equipment is a great advantage in case the above two modalities are to be employed.

On the other hand, if a patient has been ill for a short duration then it is the CT scan which is of greater utility because it is more likely to detect small subpleural haze along the hidden areas of say mediastinal reflections of pleura which might be missed on an x-ray altogether (Fig.2). It may also detect the mediastinal lymph nodes which have a strong negative prediction against the COVID-19 disease, or detect alternate pathological diagnosis of pneumonias or pulmonary infection of other etiology. The issue here would be availability of CT, its lack of portability, the possibility of contamination and spread of infection necessitating disinfection with >70% alcohol and closing the whole exposed suite for an adequate length of time, disrupting the flow of service.^6^ Therefore, a balance of pros and cons necessitates communication between the clinical and radiology care providers for optimum benefit. Thin -cut non contrast CT scan is good enough for diagnosis.

***c)Grading the disease severity:***

COVID pneumonia spreads from basal and peripheral subsegmental foci to involve, segments, lobes and the whole lung, looking like white-washed lungs. For a severely ill patient presenting at this stage, there is practically no practical advantage of either the x-rays or the ultrasound. It should, in fact, must be the CT scan to be the imaging modality of choice. CT scan objectively assesses the geographic spread of involvement and change from ground glass opacity to consolidation or fibrosis. CT scores are also developed along the scale of 0-25 based on these evolution patterns, which can further help in the stratification of the severity.

***d)Monitoring:***

Monitoring the response of treatment for resolution or progress, onset of complication like pulmonary infarcts and embolism, pleural effusion and recovery status, is again best done by CT scan. It shows not only these complications but also the residual disease in those who recover enough from the pneumonia to be discharged home. Here the technique of CT differs greatly according to the requirements. If reduction in severity and grading is to be gauged, then standard thin-cut plain CT is the answer. Mediastinal abnormalities and complications require contrast enhanced CT scan and pulmonary embolism needs a dedicated CTPA protocol. Using tailored protocols can overcome the movement artifacts and the radiation dose delivered in a CT scan.^7^

### Current situation

It is obvious that the use of different imaging modalities is defined by the clinical scenarios. This is what the various Professional Societies including the American College of Radiology, The British Society of Thoracic Imaging, the Royal College of Clinical Radiology and the Fleischner Society, are recommending.^6^, ^8^-^10^ The Radiological Society of Pakistan is fully endorsing the Fleischer Society Consensus Statement (www.radiologypakistan.org.pk).

To be cautious, Government of Pakistan has included only chest x-ray in the current management guidelines which is in keeping with our own local issues of cross infection, disinfection, logistic and infrastructural shortcomings, equipment availability and resources limitation.

New studies regarding imaging are pouring in by the hour, trying to influence and change the practice views. At the time of preparing this manuscript, the prevailing confusion among some radiologists and majority of Authorities is a result of ignoring the balance and the practical ground realities in an attempt to use more sophisticated equipment.

## CONCLUSION

This writeup describes the approach for a rational use of imaging to the best advantage in the current situation according to local resources, and restricting the spread of infection. The most practical compromise for Pakistan appears to be the use of portable digital radiography equipment, and point-of- care ultrasound; with CT scan reserved for clinical situations not explained by the above two modalities, or demanding disease stratification.
